# The introduction of video-enabled directly observed therapy (video-DOT) for patients with drug-resistant TB disease in Eswatini amid the COVID-19 pandemic – a retrospective cohort study

**DOI:** 10.1186/s12913-024-11151-4

**Published:** 2024-06-03

**Authors:** Bernhard Kerschberger, Michelle Daka, Bhekiwe Shongwe, Themba Dlamini, Siphiwe Ngwenya, Clara Danbakli, Bheki Mamba, Bongekile Nxumalo, Joyce Sibanda, Sisi Dube, Lindiwe Mdluli Dlamini, Edwin Mabhena, Esther Mukooza, Iona Crumley, Iza Ciglenecki, Debrah Vambe

**Affiliations:** 1 Médecins Sans Frontières (MSF), Mbabane, Eswatini; 2 National TB Control Programme (NTCP), Manzini, Eswatini; 3 Médecins Sans Frontières, Geneva, Switzerland

**Keywords:** Video-enabled DOT, DOT, Drug-resistant TB, COVID-19

## Abstract

**Background:**

Video-enabled directly observed therapy (video-DOT) has been proposed as an additional option for treatment provision besides in-person DOT for patients with drug-resistant TB (DRTB) disease. However, evidence and implementation experience mainly originate from well-resourced contexts. This study describes the operationalization of video-DOT in a low-resourced setting in Eswatini facing a high burden of HIV and TB amid the emergence of the COVID-19 pandemic.

**Methods:**

This is a retrospectively established cohort of patients receiving DRTB treatment during the implementation of video-DOT in Shiselweni from May 2020 to March 2022. We described intervention uptake (vs. in-person DOT) and assessed unfavorable DRTB treatment outcome (death, loss to care) using Kaplan-Meier statistics and multivariable Cox-regression models. Video-related statistics were described with frequencies and medians. We calculated the fraction of expected doses observed (FEDO) under video-DOT and assessed associations with missed video uploads using multivariable Poisson regression analysis.

**Results:**

Of 71 DRTB patients eligible for video-DOT, the median age was 39 (IQR 30–54) years, 31.0% (*n* = 22) were women, 67.1% (*n* = 47/70) were HIV-positive, and 42.3% (*n* = 30) were already receiving DRTB treatment when video-DOT became available. About half of the patients (*n* = 37; 52.1%) chose video-DOT, mostly during the time when COVID-19 appeared in Eswatini. Video-DOT initiations were lower in new DRTB patients (aHR 0.24, 95% CI 0.12–0.48) and those aged ≥ 60 years (aHR 0.27, 95% CI 0.08–0.89). Overall, 20,634 videos were uploaded with a median number of 553 (IQR 309–748) videos per patient and a median FEDO of 92% (IQR 84–97%). Patients aged ≥ 60 years were less likely to miss video uploads (aIRR 0.07, 95% CI 0.01–0.51). The cumulative Kaplan-Meier estimate of an unfavorable treatment outcome among all patients was 0.08 (95% CI 0.03–0.19), with no differences detected by DOT approach and other baseline factors in multivariable analysis.

**Conclusions:**

Implementing video-DOT for monitoring of DRTB care provision amid the intersection of the HIV and COVID-19 pandemics seemed feasible. Digital health technologies provide additional options for patients to choose their preferred way to support treatment taking, thus possibly increasing patient-centered health care while sustaining favorable treatment outcomes.

**Supplementary Information:**

The online version contains supplementary material available at 10.1186/s12913-024-11151-4.

## Background

Drug-resistant TB (DRTB) remains a major public health concern, with about 410,000 people developing the disease and only 175,650 people initiated on therapy globally in 2022 [1]. Although DRTB disease is curable, treatment success remained low at 63% [1]. Complicating the situation in Southern Africa, about 67% of TB/DRTB patients are co-infected with HIV [2], which is the main contributor to TB/DRTB-related mortality [3–6].

To increase adherence to TB therapy, directly observed therapy (DOT) has been proposed over unsupervised therapy as a key element of DRTB treatment administration [7–10]. DOT requires a person – preferably a health worker or trained lay provider – to physically observe the patient taking the medication [9]. However, in-person DOT is resource intensive (e.g. human resource requirements, out-of-pocket travel costs for patients) and a main contributor to the catastrophic costs for TB patients in low-resourced settings [11, 12]. Notably, WHO made the conditional recommendation that video-enabled DOT (video-DOT) may replace in-person DOT if digital health technologies are available and can be safely operated by health workers and patients [9]. With video-DOT, patients use a digital device (e.g. smartphone) remotely to take a video of themselves swallowing the medication, which is then either watched in real time (synchronous) or reviewed later (asynchronous) by a health worker or trained lay person [13]. Video-DOT has been mainly piloted in high-income countries and increased the proportion of verified prescribed doses taken, appeared to be programmatically feasible and cost-effective, and was acceptable to health workers and patients, while treatment outcomes remained similar to in-person DOT [10, 14–20]. However, little evidence is available from low-resourced and high HIV- and TB-burden settings [9, 13, 21], where digital health communication technologies may be most needed but remain limited given unreliable internet connectivity and possible unaffordability of smartphones and mobile data for patients [22].

Video-DOT may offer advantages when in-person DOT is impractical. For instance, video-DOT may ensure continuity of DOT during COVID-19 public health lockdowns and may also decrease the risk of COVID-19 infection that is known to increase mortality in patients co-infected with HIV and TB [23]. In 2020, Médecins Sans Frontières (MSF) and the National TB Control Programme (NTCP) of Eswatini introduced video-DOT, aiming at providing safer DRTB treatment care options during periods of high COVID-19 transmission. This is to our knowledge the first study from a low-resourced rural setting describing the operationalization of video-DOT in the face of the triple TB, HIV and COVID-19 pandemics.

## Methods

### Setting

Eswatini has a high burden of HIV (24.8% in ≥ 15 year-olds, in 2021 [24]) and TB (325 cases per 100,000 population in 2022 [1]), with 65% of TB cases co-infected with HIV [1]. The country faces high income inequality (Gini index of 54.6 in 2016) and poverty (36.1% poverty headcount ratio at USD 2.15 a day) [25]. In 2020, 107 mobile cellular subscriptions were recorded per 100 people and the average cost of 1 gigabyte of mobile internet data was USD 0.84 in 2022 [25, 26]. In 2016/17, most DRTB patients had multiple socio-economic vulnerabilities, with 55% having primary school education or lower, 83% being unemployed, 86% living in a household with an income < 74 USD, and 54% residing > 20 km away from the nearest treatment facility [27]. In Eswatini, the first case of COVID-19 was detected in March 2020 and was followed by four COVID-19 waves until December 2021 [28].

Video-DOT was piloted in the southern, predominantly rural, Shiselweni region. In 2017, it had a population of ~ 204,000, with 61% being ≥ 15 years old, and a population density of 54 per square kilometer [29].

### DRTB care

#### DRTB care

DRTB care was provided at three secondary care facilities [30]. Diagnosis was by genotypic or phenotypic testing or based on clinical grounds. Medical doctors initiated a standardized oral DRTB treatment regimen for a duration of approximately 9–20 months, and antiretroviral therapy in patients with HIV co-infection. In-person DOT was provided by a nurse at the facility or by a trained lay person at the patient’s home. Patients visited the facility each month for clinical review, laboratory follow-up tests, drug refills and adherence support. Community TB nurses provided home visits as well as phone and physical defaulter tracing. Patients could be hospitalized in one DRTB ward in case of clinical complications or adherence challenges at treatment initiation or during follow-up.

#### COVID-19 care

COVID-19 testing was performed with antigen rapid-diagnostic tests and PCR assays for DRTB patients presenting with symptoms suggestive of COVID-19 and routinely if admitted to the TB ward. Therapy for clinically uncomplicated COVID-19 included anti-pyretic medication, vitamin C, zinc and azithromycin.

#### Video-DOT

**Fig. 1:**
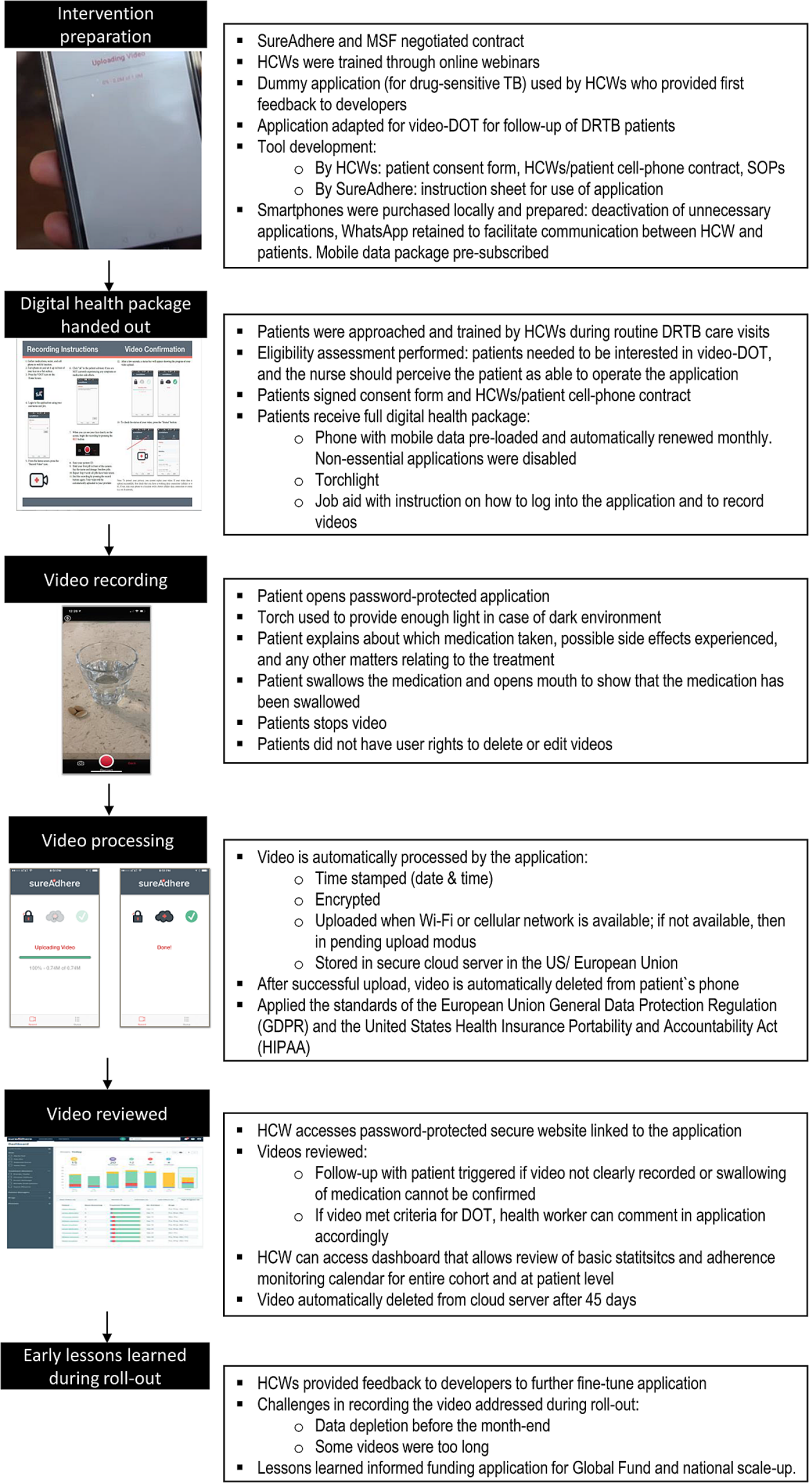
Flowchart of video directly observed therapy (VDOT) procedures DOT: directly observed therapy; DRTB: drug-resistant tuberculosis; HCWs: health care workers; SOPs: standart operating procedures; video-DOT: video-enabled directly observed therapy

Figure 1 displays the video-DOT procedures applied in Shiselweni. In summary, the SureAdhere application [31] – originally used for monitoring of drug-sensitive TB care – was adapted to allow video-DOT for patients receiving DRTB treatment. TB nurses were trained on provision of asynchronous video-DOT, and they developed the contextualized tools needed for the implementation with assistance from SureAdhere. The training of health care workers was specifically designed to facilitate the launch of video-DOT on a pilot basis. Following the completion of the pilot, the trainings were refined and expanded to support a national rollout of video-DOT. Additional details on the trainings for healthcare workers and patients can be found in the Supplementary Material.

MSF provided patients with a free smartphone package comprising a smartphone (USD 117) with the preinstalled application, a preregistered SIM card (USD 3) and a monthly reloadable voucher for mobile cellular data (USD 19/month).

Video-DOT was offered to ≥ 18-year-old patients receiving DRTB treatment in the absence of clinical danger signs (e.g., fever, pulmonary decompensation). Patients opting for video-DOT received a short practical introduction to video recording and needed to sign a consent form.

The patient-recorded videos were automatically encrypted by the application, time-stamped, uploaded to a secure cloud-based server for storage, and automatically deleted from the phone after successful upload. In case of unavailability of a cellular network, the video was temporarily stored on the phone until a connection was available.

The nurse reviewed the stored videos through a password-protected secure web interface at the MSF office. If side effects (e.g. signs of peripheral neuropathy) or other issues (e.g. emotional stress) were reported or observed (e.g. medication not properly taken), the nurse could immediately contact the patient via WhatsApp or phone call, or initiate a home visit.

### Study design

This is a retrospectively established cohort of patients receiving DRTB treatment during the implementation of video-DOT (vs. in-person DOT) in Shiselweni from May 2020 to March 2022.

### Main definitions

A DR-TB treatment case was a patient who was recorded in the DR-TB treatment register and who received any combination of second-line anti-TB drugs due to laboratory-confirmed drug-resistance or following a clinician’s empirical decision for second-line therapy.

In-person DOT was defined as a healthcare worker or trained individual directly observing the patient taking the DR-TB medication during each dose. Exposure to video-DOT was defined as a patient who was trained and registered for video-DOT and who received the smartphone package.

Enrollment into the cohort occurred at the date of video-DOT eligibility. This was the time when video-DOT became programmatically available (1 May 2020) for patients already on DRTB treatment who had an expected ≥ 3 months remaining for completion of therapy. It was the date of DRTB treatment initiation for patients starting DRTB treatment after that date until 31 December 2021.

#### Outcomes

First, uptake of video-DOT was defined as the date of the first uploaded video. Patients lacking records of video upload were assumed to be under in-person DOT.

Second, missed video upload was defined as days without a log of an uploaded video.

Third, the DR-TB program consistently utilized WHO recommended definitions to determine crude treatment outcomes [32]. For this study, the composite unfavorable treatment outcome was defined as the occurrence and date of death, treatment failure or loss to care. Patients continuing in-person or video-DOT after treatment failure were considered as retained in DRTB care until the next recorded outcome. Follow-up time was censored at the time of transfer out or the end of the observation period (database closure on 31 March 2022) for patients active on treatment. This gave all new DRTB treatment initiations enough time (3 months) to initiate video-DOT and for all observations to meet the definition of lost to care, defined as not presenting to care at the facility or no video upload for ≥ 3 months.

### Data management

DRTB treatment data were routinely extracted by a trained data clerk into an electronic DRTB database used for routine program monitoring. These data were linked with video log data from the SureAdhere platform. Records from the TB nurse complemented information on COVID-19 co-infections.

### Statistical analysis

Analyses were performed with Stata 17 [33]. Baseline data were described using frequency statistics and proportions.

#### Video-DOT uptake and unfavorable outcome

Crude Kaplan-Meier estimates and plots describe time from video-DOT eligibility to intervention uptake and to the composite unfavorable treatment outcome. Associations between baseline characteristics and time to these outcomes were assessed in Cox-regression analyses, using the backward selection method to fit the final multivariable model.

#### Video-DOT-related statistics

Patient-level adherence to video-DOT was estimated by calculating the median fraction of expected doses observed (FEDO) during video-DOT time as similarly applied in other studies [14, 34]. FEDO was obtained by dividing the total number of video uploads – a proxy for treatment dose taken – per patient by the number of expected video uploads (two per day) during treatment. Video-DOT treatment time was measured from the date of uptake of video-DOT to the treatment outcome date and was adjusted for hospitalization by subtracting the number of hospitalization days from the numerator assuming that in-person DOT was practiced. To assess associations between baseline factors and the rate of missed video uploads, we built negative binomial regression models that were adjusted for hospitalization.

#### COVID-19

Time series plots were used to display the evolution of the COVID-19 pandemic in Eswatini vs. timing of uptake of video-DOT, follow-up care and outcomes. Country-specific COVID-19 data (daily cases of and deaths from COVID-19 and the stringency index) were obtained online [35]. The stringency index estimates on a 0–100 scale the lockdown strictness and is a measure of the composite severity of nine government COVID-19 public health policies [36]. The population adjusted 7-day moving average of COVID-19 cases (per 1 million population) and deaths (per 10 million population) were calculated by dividing crude daily numbers by Eswatini population estimates.

### Ethics

All methods were carried out in accordance with the Declaration of Helsinki. The need for informed consent was waived by the ethics committee of the Eswatini Health and Human Research Review Board (EHHRRB) because of the retrospective nature of the study. This research fulfilled the exemption criteria set by the Institutional Médecins Sans Frontières Ethics Review Board (ERB) for a posteriori analyses of routinely collected clinical data and thus did not require MSF ERB review. It was conducted with permission from Medical Director, Operational Center Geneva Médecins Sans Frontières.

## Results

### Baseline characteristics

Of 71 DRTB treatment cases eligible for video-DOT (Table 1), 30 (42.3%) were already receiving DRTB treatment at the time when video-DOT became available. The median age was 39 (interquartile range [IQR] 30–54) years, 31.0% (*n* = 22) were women, 40.8% (*n* = 29) lived in a partnership, and 60.6% (*n* = 43) were unemployed. Thirteen (18.3%) and 10 (14.1%) patients reported alcohol consumption and smoking, respectively. Six (8.7%) patients had diabetes mellitus, 47 (67.1%) lived with HIV, and the median body mass index (BMI) was 20.4 (IQR 18.0–23.4) kg/m^2^. Most patients had bacteriologically confirmed DRTB disease (*n* = 68; 97.1%) and 34 (47.9%) reported past TB treatment. About half of patients (*n* = 34; 47.9%) became eligible for video-DOT when the COVID-19 stringency index was ≥ 0.75, the median 7-day moving average of new COVID-19 cases per 1 million population was 9 (IQR 9–41) and the median 7-day moving average of COVID-19 deaths was 0 (IQR 0–9) per 10 million population.

**Table 1:** Baseline characteristics of patients treated for DRTB disease and monitored under the in-person DOT or video-DOT approach in Shiselweni, Eswatini

	In-person DOT (*n* = 34)		Video-DOT (*n* = 37)		Entire cohort (*n* = 71)		*p*-value^2^
	No	(%)	No	(%)	No	(%)	
**DRTB treatment status**							
On treatment	8	(23.5)	22	(59.5)	30	(42.3)	0.002
New treatment initiation	26	(76.5)	15	(40.5)	41	(57.7)	
**Age; years**							
18 to 59	25	(73.5)	34	(91.9)	59	(83.1)	0.039
≥ 60	9	(26.5)	3	(8.1)	12	(16.9)	
**Sex**							
Male	26	(76.5)	23	(62.2)	49	(69.0)	0.193
Female	8	(23.5)	14	(37.8)	22	(31.0)	
**Marital status**							
Partnership	17	(50.0)	12	(32.4)	29	(40.8)	0.132
Single	17	(50.0)	25	(67.6)	42	(59.2)	
**Employment status**							
Unemployed	21	(61.8)	22	(59.5)	43	(60.6)	0.843
Employed or student	13	(38.2)	15	(40.5)	28	(39.4)	
**Alcohol**							
No	27	(79.4)	31	(83.8)	58	(81.7)	0.634
Yes	7	(20.6)	6	(16.2)	13	(18.3)	
**Smoker**							
No	26	(76.5)	35	(94.6)	61	(85.9)	0.028
Yes	8	(23.5)	2	(5.4)	10	(14.1)	
**BMI**^1^; **kg/m**^**2**^							
≥ 18.5 to < 25	17	(53.1)	21	(56.8)	38	(55.1)	0.762
≥ 25	15	(46.9)	16	(43.2)	31	(44.9)	
**Diabetes mellitus** ^1^							
No	28	(87.5)	35	(94.6)	63	(91.3)	0.297
Yes	4	(12.5)	2	(5.4)	6	(8.7)	
**HIV status** ^1^							
Negative	13	(39.4)	10	(27.0)	23	(32.9)	0.271
Positive	20	(60.6)	27	(73.0)	47	(67.1)	
**Past TB treatment**							
No	14	(41.2)	20	(54.1)	34	(47.9)	0.278
Yes	20	(58.8)	17	(45.9)	37	(52.1)	
**Bacteriologically confirmed TB** ^1^							
No	0	(0.0)	2	(5.4)	2	(2.9)	0.175
Yes	33	(100.0)	35	(94.6)	68	(97.1)	
**COVID-19 stringency index**							
0 to < 0.75	24	(70.6)	13	(35.1)	37	(52.1)	0.003
≥ 0.75	10	(29.4)	24	(64.9)	34	(47.9)	
**COVID-19 cases per 1 million population**	17	(9–50)	9	(9–23)	9	(9–41)	0.122
**COVID-19 deaths per 10 million population**	9	(0–17)	0	(0–9)	0	(0–9)	0.024
**Distance to nearest DOT center, km**							
0 to < 1	NA		2	(5.4)	NA		
≥ 1 to < 5	NA		12	(32.4)	NA		
≥ 5 to < 10	NA		13	(35.1)	NA		
≥ 10	NA		10	(27.0)	NA		

aHR: adjusted hazard ratio; BMI: body mass index; cHR: crude hazard ratio; DOT: directly observed therapy; DRTB: drug-resistant TB; km: kilometers; video-DOT: video directly observed therapy

^1^ The variables BMI and diabetes mellitus each had 2.8% (*n* = 2) of values missing, and HIV status and bacteriologically confirmed TB each had 1.4% (*n* = 1) values missing

^2^ Differences between categorical variables were assessed with the Pearson’s chi-squared test, and those between medians with the Wilcoxon rank-sum test

### Uptake of video-DOT

**Fig. 2:**
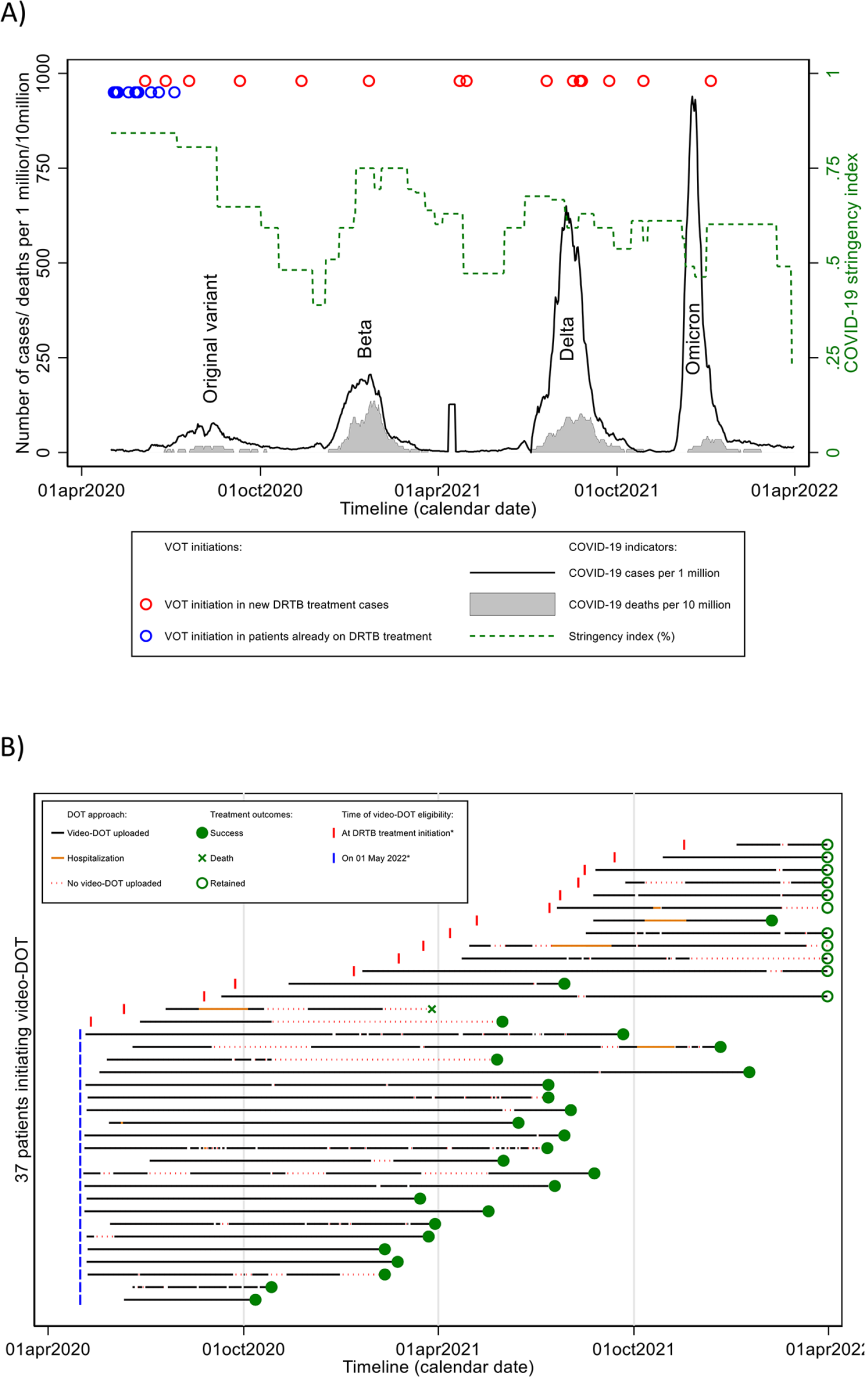
Timeplots displaying the evolution of COVID-19 and the implemenation of video-enabled directly observed DRTB care Plot A displays the evolution of the COVID-19 pandemic and the COVID-19 stringency index in Eswatini, and times when video-DOT was initiated in patients already on DRTB treatment and in patients newly initiated on DRTB therapy. Plot B displays times when patients became eligible for video-DOT, follow-up video-DOT care times (on video-DOT, hospitalization, non-observed therapy) and health outcome at the time of database closure

Of 37 (52.1%) patients initiating video-DOT, most started immediately before or during the first wave of COVID-19 that coincided with high levels of COVID-19 stringency index and the beginning of programmatic availability of video-DOT (Fig. 2). During the early implementation period, most video-DOT initiations were by patients already receiving DRTB treatment, whereas it was solely patients newly initiating DRTB treatment during later implementation periods (Fig. 2). Patients initiating video-DOT tended to be younger (37 [IQR 29–45] vs. in-person DOT: 44 [IQR 32–60] years; *p* = 0.057), nonsmokers (5.4% vs. 23.5%; *p* = 0.028), and more likely to become eligible for video-DOT during time periods when the COVID-19 stringency index was ≥ 0.75 (64.9% vs. 29.4%; *p* = 0.003) and the median daily COVID-19 deaths were lower (0 [IQR 0–9] vs. 9 [0–17]; *p* = 0.024). No other obvious differences in baseline characteristics were detected. For patients using video-DOT, the median distance to the nearest DOT facility was 6 (IQR 3–6) km, with the shortest being < 0.5 km and longest 20 km.

**Fig. 3:**
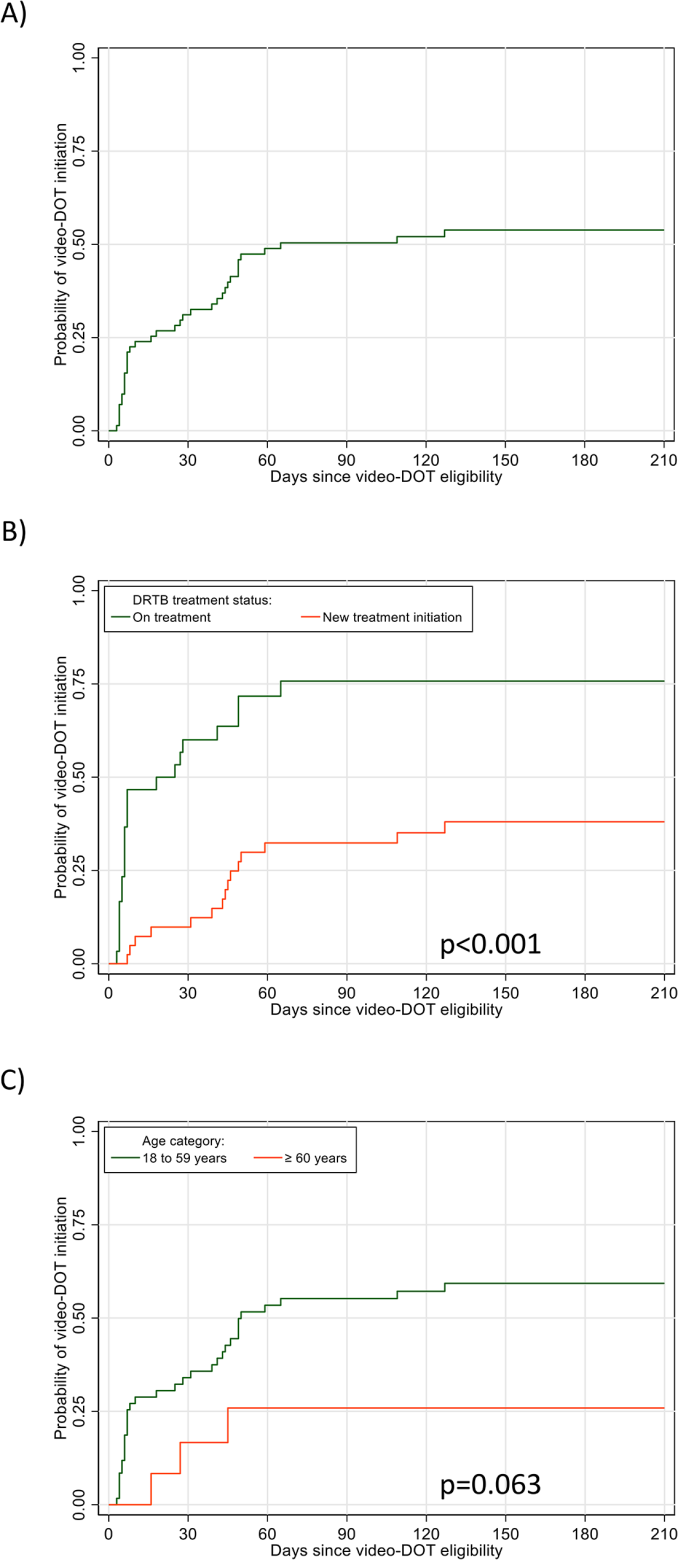
Kaplan-Meier plots of (**A**) overall uptake of video-DOT from time of video-DOT eligibility, and uptrake by (**B**) DRTB treatment status and (**C**) age groups in Shiselweni, Eswatini

The crude cumulative probability (Kaplan-Meier estimate) of video-DOT initiation was 0.21 (95% confidence interval [CI] 0.13–0.33) at 7 days after eligibility for video-DOT, increasing to 0.54 (95% CI 0.43–0.66) at 6 months. Initiations tended to be lower for new DRTB treatment cases and for patients aged ≥ 60 years (see Fig. 3), and higher for time periods of COVID-19 stringency index ≥ 0.75 (Table 2).

**Table 2:** Univariate and multivariable associations between baseline factors and time to initiation of video-DOT in Shiselweni, Eswatini

	Univariate analysis (*n* = 71)^1^		Multivariable analysis (*n* = 71)^1^	
	cHR	(95% CI)	aHR	(95% CI)
**DRTB treatment status**				
On treatment	1		1	
New treatment initiation	0.27	(0.14–0.53)	0.24	(0.12–0.48)
**Age; years**				
18 to 59	1		1	
≥ 60	0.35	(0.11–1.13)	0.27	(0.08–0.89)
**Sex**				
Male	1			
Female	1.58	(0.81–3.08)		
**Marital status**				
Partnership	1			
Single	1.78	(0.89–3.54)		
**Employment status**				
Unemployed	1			
Employed or student	1.03	(0.53–1.98)		
**Alcohol**				
No	1			
Yes	0.80	(0.33–1.92)		
**Smoker**				
No				
Yes	0.28	(0.07–1.16)		
**BMI**^1^; **kg/m**^**2**^				
≥ 18.5 to < 25	1			
≥ 25	0.85	(0.44–1.64)		
**Diabetes mellitus** ^1^				
No	1			
Yes	0.51	(0.12–2.14)		
**HIV status** ^1^				
Negative	1			
Positive	1.50	(0.73–3.11)		
**Past TB treatment**				
No	1			
Yes	0.59	(0.31–1.12)		
**Bacteriologically confirmed TB** ^1^				
No	1			
Yes	0.55	(0.13–2.33)		
**COVID-19 stringency index**				
0 to < 0.75	1			
≥ 0.75	3.38	(1.71–6.69)		
**COVID-19 cases per 1 million population**	1.00	(1.00–1.00)		
**COVID-19 deaths per 10 million population**	0.99	(0.98–1.01)		

aHR: adjusted hazard ratio; BMI: body mass index; cHR: crude hazard ratio; DOT: directly observed therapy; DRTB: drug-resistant TB; video-DOT: video directly observed therapy

^1^ The variables BMI and diabetes mellitus each had 2.8% (*n* = 2) of values missing, and HIV status and bacteriologically confirmed TB each had 1.4% (*n* = 1) missing values. Multiple imputation by chained equation was applied to account for missing values in regression analysis. Cox proportional hazards models were built with time zero defined as the time of eligibility for the video-DOT interventions, which was 1 May 2020 for patients already on DRTB treatment or the date of DRTB treatment initiation for patients starting DRTB therapy during the roll-out of the video-DOT approach

Multivariable analysis (Table 2) showed that the likelihood of initiation of video-DOT remained lower for new DRTB patients (adjusted hazard ratio [aHR] 0.24, 95% CI 0.12–0.48) and those aged ≥ 60 years (aHR 0.27, 95% CI 0.08–0.89).

### Video-DOT indicators

Overall, 20,634 videos were uploaded with a median number of 553 (IQR 309–748) videos per patient. The median time from recording to video upload was 3 (IQR 0–49) minutes. The median FEDO adjusted for hospitalization was 92% (IQR 84–97%). Of six patients with a FEDO < 80%, two had treatment success, one died and three were still on treatment at end of study. Only older age (≥ 60 years) lowered the risk (adjusted incidence risk ratio 0.07, 95% CI 0.01–0.51) of days without uploaded videos in univariate and multivariable regression analysis (Table 3).

**Table 3:** Univariate and multivariable associations between baseline factors and number of days without recorded video uploads in Shiselweni, Eswatini

	Univariate analysis (*n* = 37)^1^		Multivariable analysis (*n* = 37)^1^	
	cIRR	(95% CI)	aIRR	(95% CI)
**DRTB status at eligibility**				
On treatment	1			
New treatment	1.44	(0.51–4.08)		
**Age; years**				
18 to 59	1		1	
≥ 60	0.07	(0.01–0.51)	0.07	(0.01–0.51)
**Sex**				
Male	1			
Female	1.11	(0.38–3.22)		
**Marital status**				
Partnership	1			
Single	2.22	(0.75–6.61)		
**Employment status**				
Unemployed	1			
Employed or student	1.91	(0.68–5.35)		
**Alcohol**				
No	1			
Yes	0.77	(0.19–3.10)		
**Smoker**				
No	1			
Yes	1.22	(0.13–11.78)		
**BMI**^**1**^; **kg/m**^**2**^				
≥ 18.5 to < 25	1			
≥ 25	1.57	(0.56–4.41)		
**Diabetes mellitus** ^**1**^				
No	1			
Yes	3.70	(0.41–33.39)		
**HIV status** ^**1**^				
Negative	1			
Positive	0.91	(0.28–2.89)		
**Past TB treatment**				
No	1			
Yes	1.32	(0.47–3.71)		
**Bacteriologically confirmed TB** ^**1**^				
No	1			
Yes	2.67	(0.27–26.83)		
**COVID-19 stringency index**				
0 to < 0.75	1			
≥ 0.75	0.75	(0.26–2.20)		
**COVID-19 cases per 1 million population**	1.00	(1.00–1.00)		
**COVID-19 deaths per 10 million population**	1.01	(0.99–1.03)		

aIRR: adjusted incidence risk ratio; BMI: body mass index; cIRR: crude incidence risk ratio; DOT: directly observed therapy; DRTB: drug-resistant TB; video-DOT: video directly observed therapy

^1^ The variables BMI and diabetes mellitus each had 2.8% (*n* = 2) of values missing, and HIV status and bacteriologically confirmed TB each had 1.4% (*n* = 1) missing values. Multiple imputation by chained equation was applied to account for missing values in regression analysis. Negative binomial regression models were built as there was evidence of overdispersion of the count variable (missed video uploads per patient)

### COVID-19

Two COVID-19 cases were diagnosed under video-DOT vs. one under in-person DOT. All cases were men aged 35–62 years, nonsmoking, living with HIV, without diabetes mellitus, and with BMI 18.6–24.4 kg/m^2^. Their COVID-19 vaccination status was unknown. All patients recovered from COVID-19 and remained active on DRTB treatment at end of study.

### Treatment outcomes

Overall, 38 (53.5%) patients had treatment success (1 completed, 37 cured), and 28 (39.4%) were still active on therapy at end of study. Five (7.0%) patients had an unfavorable treatment outcome (3 deaths, 2 lost to care).

**Fig. 4:**
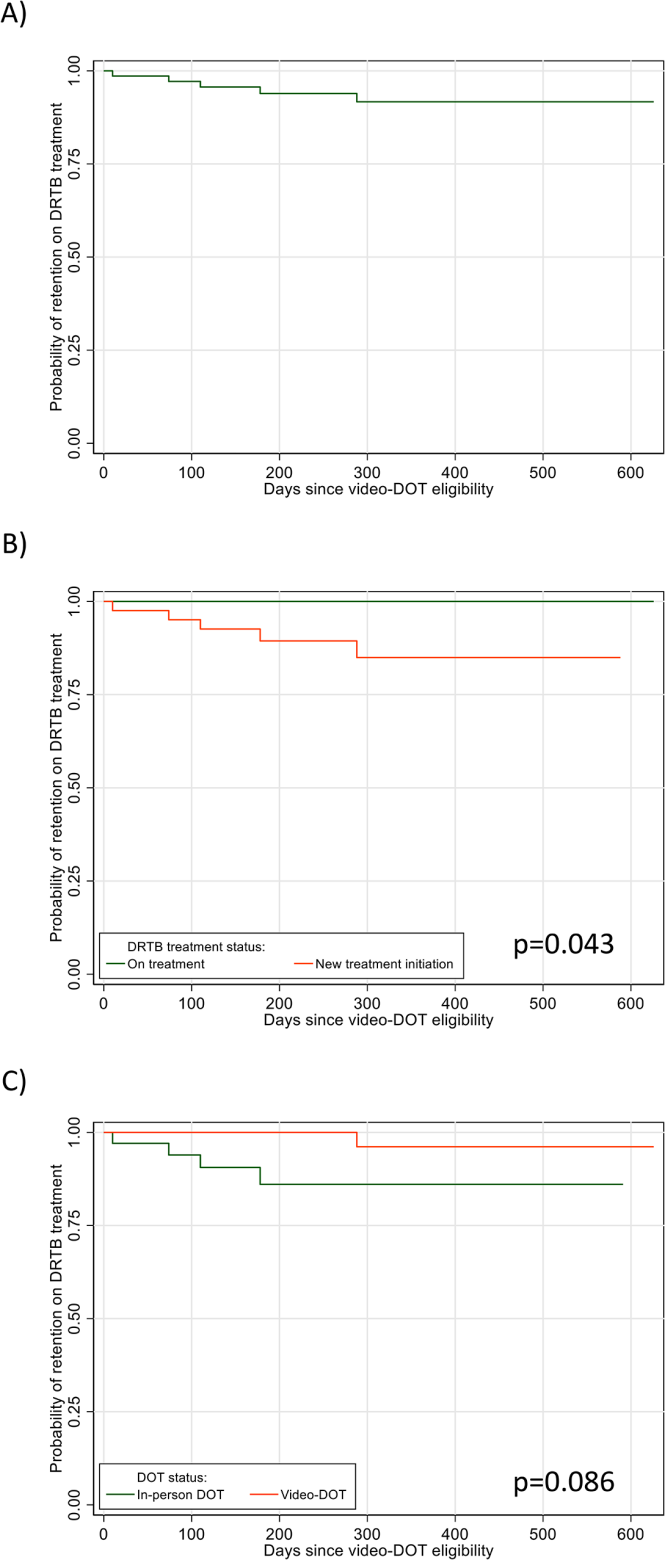
Kaplan-Meier plots of retention on DRTB treatment from time of eligiblity for video-DOT, (**A**) overall and by (**B**) DRTB treatment status and (**C**) age groups in Shiselweni, Eswatini

The crude cumulative probability of an unfavorable treatment outcome was 0.08 (95% CI 0.03–0.19) (Fig. 4). Patients already on DRTB treatment (*p* = 0.043) and followed under video-DOT (*p* = 0.086) tended to experience less unfavorable outcomes (Fig. 4). However, univariate and multivariable analyses did not detect any obvious associations between baseline factors and time to unfavorable treatment outcome.

## Discussion

Although the COVID-19 pandemic negatively affected the allocation of resources and delivery of TB care globally [37], the pandemic provided an opportunity for the introduction of digital health interventions [37, 38]. We introduced video-DOT under routine conditions for patients treated for DRTB disease in this low-resourced, high HIV-burden setting amid the emergence of COVID-19. About half of our DRTB patients chose video-DOT over in-person DOT, with high rates of treatment adherence and favorable treatment outcomes achieved.

### Interpretation of findings

Video-enabled DOT in well-resourced settings showed higher acceptance than in-person DOT by patients and health workers [14, 16, 19, 39]. In our context, half of DRTB patients (52.1%) chose video-DOT, with younger age and existing receipt of DRTB treatment when the intervention became available being the main predictors of uptake. Older patients may face digital inequalities regarding skills in digital technologies. Health workers reported that older patients found using video-DOT childish or complicated. In addition, there may be other factors associated with uptake that we did not measure such as stigma perceived by patients and the efficacy of training on the use of video-DOT [40]. Notably, the DRTB program applied a patient-centered approach providing patients with a choice between in-person DOT and video-DOT rather than being prescriptive, thus supporting a differentiated care package adapted to the patient’s ability and willingness regarding digital health support.

Interruptions of video-DOT during treatment provision were not uncommon. Some patients transitioned to in-person DOT temporarily during hospitalizations or permanently due to adherence or logistic issues. A study from Uganda using video-DOT for patients with drug-sensitive TB showed that the top three reasons for interruptions were practical/technical obstacles in using the application, battery not being charged and application errors [34]. Non-technical factors included lack of TB medication, non-privacy and forgetting to record the video [34]. Importantly, video-DOT interruption does not mean that treatment doses were missed as long as medication intake was through in-person DOT or self-administration, with the latter possibly being as high as 59% for drug-sensitive TB therapy [34]. Importantly, the FEDO was high (92%) in our study, suggesting high levels of adherence to therapy, and comparable to a study from the US enrolling drug-sensitive and drug-resistant TB cases (93%) [14] and slightly higher than in a study from Uganda (85%) enrolling patients with drug-sensitive TB [34].

The probability of an unfavorable treatment outcome – as measured from the time of study eligibility – was low overall. However, our estimates should not be compared with DRTB cohorts that measure treatment success in new treatment initiations. Our study enrolled both patients already on DRTB treatment and newly initiated patients to better describe the video-DOT intervention and to avoid a too-small sample size that would have reduced our ability to obtain meaningful estimates. Nevertheless, crude analysis showed a tendency for patients using video-DOT to be more often retained in DRTB care, possibly explained by higher adherence to therapy because of fewer barriers to treatment taking or because of other unmeasured risk factors that may increase the likelihood of an unfavorable treatment outcome for in-person DOT (e.g. comorbidities). A recent systematic review suggested that different approaches to DOT (e.g. in-person, by video) vs. self-administered therapy and DOT delivered at community level (vs. clinic) resulted in better intermediate (e.g. sputum conversion) and final health outcomes (e.g. treatment success) [10]. Video-DOT could be considered as combining these two approaches, supported by evidence that patients under video-DOT have similar treatment outcomes to patients followed by in-person DOT [10]. Finally, patients already receiving DRTB treatment at the time when video-DOT became available tended to have higher retention in care, possibly explained by survival bias as patients who died or became lost to care before video-DOT were excluded from analysis, thus retaining healthy survivors only. Notably, no significant predictors were identified after adjustment for covariate factors.

### Findings in context

While not evaluated in this study, video-DOT could provide supplementary benefits in the management of drug-resistant TB care. Firstly, although our intervention was nurse-controlled, some routines could be task-shifted to lower healthcare cadres, thus freeing nurse time for other activities. For instance, after completion of the pilot, a lay HIV/TB adherence counselor was trained to review uploaded videos and support adherence interventions in tandem with the nurse. Secondly, the flexibility of video-DOT enables health workers to review videos from any location with internet access and at various times, facilitating the incorporation of this method into their regular work schedules. However, if video reviews are delayed (e.g. over weekends), alternative communication methods should be available for patients experiencing severe side effects. In our context, patients could directly call the nurse at any time for reporting of side effects and requesting support. Thirdly, less nurse human resource time was probably required, with one nurse providing video-DOT for patients in the entire region vs. several TB nurses providing in-person DOT or training for community-based volunteers providing in-person DOT.

Other considerations are equity in access to digital health technologies. Video-DOT requires patients to afford a smartphone, internet access and mobile data. Notably, suboptimal smartphone ownership has been identified in better resourced settings as a possible barrier to digital health interventions, possibly perpetuating health disparities [41]. In addition, we provided free smartphones and internet data bundles to all patients to reduce structural barriers in our setting. As for instance, the costs of 1 gigabyte of mobile internet was approximately 1% of the monthly household income of DR-TB affected households [25–27]. Cost savings, however, may be feasible by using the patient’s own smartphone if available or lending one to patients as applied in a study in Uganda [34] and during the scale-up of video-DOT in Eswatini in 2022.

Considerations about data security, privacy and confidentiality are other important considerations before introduction of digital health interventions. We used a pre-established application that enabled users to upload encrypted videos onto a US-based secure server with recorded videos automatically deleted from the patient’s phone and server in due time. Compliance with local and international data regulations and laws may ensure patients’ and health workers’ confidence in this technology and reduce the risk of data breaches.

Video-DOT may offer opportunities for integration of care provision for other diseases. Although we lacked data, some patients probably had non-communicable comorbidities such as hypertension and diabetes mellitus. Thus, broadening the digital care approach may not only provide a more holistic treatment experience but also increase quality of care and overall health outcomes.

### Limitations

We did not assess costs and cost-effectiveness. Although a study from a high-income country suggested the cost-effectiveness of video-DOT during the pandemic [42], the cost-benefit ratio may vary by high- vs. low-resourced programmatic settings and population targeted. Cost-effectiveness assessments from different contexts are warranted to inform funding and health policy decisions. In addition, this study exclusively examines the quantitative aspects of video-DOT. However, incorporating patients’ and healthcare workers’ perspectives is crucial for a better understanding of the acceptance and patient-level benefits of this intervention, thereby better guiding its wider implementation.

Some patients circled in and out of video-DOT. Data on reasons for interrupting video-DOT temporarily (e.g. hospitalization) or permanently (e.g. structured discontinuation by health workers) was incomplete. Although our analysis adjusted for hospitalization, video-DOT adherence would likely be higher if these reasons were fully taken into account.

Our program targeted an adult rural population affected by poverty and high rates of HIV co-infection. Notably, other vulnerable populations affected by TB may also benefit, including drug users, and video-DOT has been used for drug-sensitive TB in resource-poor settings [34].

A strength of the study was its implementation in a routine DRTB care setting amid an aggravating COVID-19 pandemic. Despite these challenges, video-DOT appeared programmatically feasible, and lessons learned informed the NTCP’s funding application for the Global Fund, resulting in the national expansion of video-DOT since mid-2022. Finally, this study contributes to evidence of real-world feasibility of video-DOT in DRTB patients at a time when the public health threat of TB may increase after the COVID-19 pandemic. It shows that video-enabled treatment approaches are not only feasible in drug-sensitive TB programs from low-resourced settings [34] but also for patients living with DRTB disease facing economic hardships.

## Conclusions

Digital health interventions are increasingly used to support the delivery of health care. We utilized video-DOT as an additional choice to in-person DOT for DRTB treatment administration in a rural high HIV-burden setting amid the COVID-19 pandemic. Uptake of video-DOT was reasonable, with high rates of adherence and favorable treatment outcomes achieved. Video-DOT could be part of a differentiated care package with potential to increase patient-centeredness by expanding choices in DRTB care.

### Electronic supplementary material

Below is the link to the electronic supplementary material.

Supplementary Material 1

## Data Availability

The datasets used and analyzed during the current study are available from the corresponding author on reasonable request.

## Abbreviations

aHR: Adjusted hazard ration
DOT: Drectly observed therapy
DRTB: Drug-resistant Tuberculosis
FEDO: Fraction of expected doses observed
IQR: Interquartile range
MSF: Médecins sans Frontières
NTCP: National TB Control Programme
TB: Tuberculosis
WHO: World Health Organization
