# Supplementary material for: The introduction of video-enabled directly observed therapy (video-DOT) for patients with drug-resistant TB disease in Eswatini amid the COVID-19 pandemic – a retrospective cohort study
Source: BMC Health Serv Res. 2024 Jun 3;24:699. doi: 10.1186/s12913-024-11151-4 (PMC11145825; doi:10.1186/s12913-024-11151-4)
Supplement: Supplementary file 1 — Supplementary Material 1 [file 12913_2024_11151_MOESM1_ESM.docx]

**Supplementary Material**

**Study title: The introduction of video-enabled directly observed therapy (video-DOT) for patients with drug-resistant TB disease in Eswatini amid the COVID-19 pandemic – a retrospective cohort study**

**Trainings for healthcare workers**

To support the expansion of video-DOT, a comprehensive national training session was held over one and a half days. The training was for healthcare workers involved in DRTB care and included doctors, nurses, and trained lay providers from all DRTB treatment sites in Eswatini. The purpose was to equip them with a thorough understanding of the video-DOT application and its standard operating procedures. Key topics covered included the principles of video-DOT, patient eligibility criteria, and the full enrolment process including patient consent and the cell phone contract terms. Participants were also trained on the practical aspects on how to set up patient profiles, connect patient cell phones to the application, and handle video recordings including their upload, review, and analysis.

Following the initial training, refresher sessions lasting half a day were held as part of supportive supervision visits to DRTB treatment sites. These practical sessions reinforced concepts learned during the national training. Activities included using patient chronic care files to create profiles for consenting video-DOT enrolees, conducting test video recordings, and ensuring proper treatment verification through video review. The refresher training also provided participants with essential resources, including SureAdhere platform manuals, video-DOT standard operating procedures, consent forms, patient instructions and guide sheets, and cell phone contracts, to facilitate the effective implementation of video-DOT.

**Training procedures for patients**

After completing the consent procedures (including signing the patient consent form and agreeing to the terms of the cell phone contract), a unique patient profile was created on the SureAdhere application through the use the facility video-DOT laptop preloaded with data including the specific treatment regimen the patient was following. Utilizing a cell phone preloaded with data and equipped with the SureAdhere application, the healthcare worker guided the patient through the process step by step, preparing them for daily dose treatment adherence monitoring. This preparation involved gathering their medication and water, logging into the application, recording videos of themselves taking the medication, and ensuring that the video showed them taking and swallowing the medication, followed by a view of their mouth after swallowing, as well as the video upload process. Patients were also taught how to verify whether their videos were successfully uploaded. Each patient received a copy of the instruction sheet for video recording, which served as a reference until they became comfortable with the process and the SureAdhere application. The training sessions lasted between 10-15 minutes, varying with each patient's comfort level with cell phones and their ability to understand the steps within the application. In instances where a patient sent unsatisfactory videos, such as those not clearly showing them swallowing the tablets or failing to open their mouth after swallowing, or videos that were too long, the healthcare worker would call the patient to provide feedback on their performance and instructions for improvement. Should these issues persist, the healthcare worker would then provide a refresher training session, revisiting the entire process with the patient.
